# A coarse-grained molecular dynamics investigation of the role of mineral arrangement on the mechanical properties of mineralized collagen fibrils

**DOI:** 10.1098/rsif.2022.0803

**Published:** 2023-01-25

**Authors:** Mahdi Tavakol, Ted J. Vaughan

**Affiliations:** Biomedical Engineering and Biomechanics Research Centre, School of Engineering, College of Science and Engineering, University of Galway, Galway, Ireland

**Keywords:** bone biomechanics, mineralized collagen fibrils, mechanical properties, coarse-grained molecular dynamics

## Abstract

Mineralized collagen fibrils (MCFs) comprise collagen molecules and hydroxyapatite (HAp) crystals and are considered universal building blocks of bone tissue, across different bone types and species. In this study, we developed a coarse-grained molecular dynamics (CGMD) framework to investigate the role of mineral arrangement on the load-deformation behaviour of MCFs. Despite the common belief that the collagen molecules are responsible for flexibility and HAp minerals are responsible for stiffness, our results showed that the mineral phase was responsible for limiting collagen sliding in the large deformation regime, which helped the collagen molecules themselves undergo high tensile loading, providing a substantial contribution to the ultimate tensile strength of MCFs. This study also highlights different roles for the mineralized and non-mineralized protofibrils within the MCF, with the mineralized groups being primarily responsible for load carrying due to the presence of the mineral phase, while the non-mineralized groups are responsible for crack deflection. These results provide novel insight into the load-deformation behaviour of MCFs and highlight the intricate role that both collagen and mineral components have in dictating higher scale bone biomechanics.

## 1. Introduction

Bone is a composite material with a hierarchical structure that facilitates various roles in the body, including supporting locomotion, protecting vital organs and acting as a mineral reservoir [1]. At the lowest level of bone hierarchy, collagen molecules and hydroxy apatite (HAp) crystals are assembled into mineralized collagen Fibrils (MCFs) [2]. Even though higher-order structures of bones may differ, MCFs can be considered a universal building block of bone tissue [3] and are present across different bone types, species and even in other collagenous mineralized tissues such as dentine [4]. Collagen molecules in MCFs form a staggered arrangement, which gives rise to both high- and low-density collagen regions [5,6]. This feature has been described by the Hodge–Petruska model [7] for collagen, with these regions commonly termed overlap and gap regions, respectively. For many years, it was thought that the HAp mineral phase of bone was concentrated in these gap regions [8–10]. However, bone mineral volume measurements have demonstrated that there is insufficient space available in these regions to accommodate the overall mineral content in the tissue [11]. Two different solutions for this conflict are proposed in the literature, whereby the mineral extends into the overlap region (intrafibrillar) and/or into the extrafibrillar space, with experimental studies in support of each idea [12–18]. However, few studies have considered the mechanical implications of the mineral residing in the intrafibrillar or extrafibrillar spaces.

Several studies have sought to experimentally characterize the mechanical response of both mineralized and non-mineralized collagen fibrils [19–25], although there has been a wide range of properties reported. Using a range of different techniques, the reported tensile strengths of these structures have varied between 39 and 1000 MPa, while fracture strains have ranged from 5.96%–106.55% [19–25]. The wide variation in mechanical properties has arisen from the type of collagen fibrils being studied, which have been sourced from antler bone [21], tendon [20,22,23] and skin [19]. Across these different fibrillar systems, the amount of mineral and its distribution can vary substantially, which makes the comparison of different experimental studies difficult. In the case of non-mineralized collagen fibrils, Gentleman *et al*. measured the mechanical properties of non-mineralized collagen from rat tail tendon showing ultimate tensile strength (UTS) and fracture strains of 170 MPa and 23%, respectively [20]. With increasing mineralization, there has been a tendency for the fracture strains to reduce in MCFs. For example, Hang and Barber conducted atomic force microscopy experiment on antler bone [21] and determined that the fracture stress and strains were 82–185 MPa and 5.14–6.72%, respectively. While this data has commonly been used as an input for computational models of bone tissue [26–28], computational models of isolated MCFs have shown fracture strains much larger than this [29]. While this may be a result of different configurations of minerals throughout the MCF, it is important to note that it is extremely difficult to isolate and test MCFs, and generally much of our understanding of MCF behaviour from experimental studies would also include contributions from the extrafibrillar matrix of bone, or from the tendon inter-fascicular matrix. To address this, several studies have sought to use computational methods to examine the behaviour of idealized representations of MCFs.

Several theoretical [26,30–34] and computational models [28,35–44] have been developed to predict the effective properties of mineralized tissues. These models have considered a range of configurations, but have generally assumed a staggered composite model of HAp minerals and collagen to predict the effective properties of the tissue through theoretical or numerical means. For the most part, these models predict similar behaviour to the original staggered model of Jäger & Fratzl [32], who showed that the mineral phase was largely responsible for tissue stiffness by carrying tensile stresses, with collagen components enabling stress transfer between minerals through shear loading. Although, several studies have also explicitly considered the important relative roles of extra- and intra-fibrillar mineralization in determining the effective properties of the tissue [44]. While these models have provided insight into the effective properties of mineralized tissues, they have assumed continuum representations of the constituents and generally only considered small-deformation behaviour. More recently, discrete approaches have been developed to investigate the behaviour of collagen and mineral configurations at a molecular level, examining both elastic and viscoelastic properties of smaller collagen-like peptides, whereby the initial structure was built by computational tools such as BuScr [29]. For instance, Gautieri *et al*. studied the viscoelastic behaviour of single collagen-like peptides through molecular simulation of the creep test [45]. Since the calculated viscosity value for a single collagen molecule was several orders of magnitude lower than the corresponding value of collagen fibril, it was deduced that the role of molecular sliding and water molecules was significant in the fibril response. Ghodsi & Darvish [46] also studied the viscoelastic response of lysine–lysine cross-link between two collagen segments through steered molecular dynamics (SMD) simulations and developed a model for the viscoelastic behaviour of the cross-link. In't Veld and Stevens studied the tensile strength and Young's modulus of collagen molecules and also simulated the separation of collagen helices to measure its internal stability [47]. While these initial studies have enabled novel insight into the mechanical properties of a single collagen molecule and interactions between collagen-like peptides, they do not give a realistic representation of the MCF due to their simplistic collagen model that ignores collagen–collagen interactions.

The experimental discovery of the collagen structure by Orgel *et al*. [48] provided a more realistic representation of collagen molecules, which has been incorporated into more recent computational studies. For example, Nair *et al*. [3] investigated the tensile loading of a unit cell structure, where it was shown that minerals were primarily responsible for carrying external loading, while the deformation was mainly exerted into the collagen fibrils. Using a similar model, Gautieri *et al*. [49] illustrated a significant role for water molecules in the mechanical response of collagen to tensile loading under small deformation. In the early phase of deformation, a lower tensile modulus was observed due to initial collagen straightening, while molecule stretching in the subsequent stage led to stiffening behaviour. Gautieri and co-workers also investigated collagen sliding by pulling a collagen molecule [50] in dry hexagonally arranged micro-fibril. Here, deformation was found to be mainly due to collagen molecule stretching, while the presence of water was observed to play a lubricating role to facilitate the collagen sliding. In these studies, due to the prohibitive costs of modelling the full fibril, a unit-cell or a micro-fibril composed of six collagen molecules from this structure was modelled, with the deformation limited to the elastic regime.

More recently, a mesoscopic model for the simulation of the collagen structure has been developed, which has enabled a computationally feasible tool for studying full collagen fibrils. Buehler *et al.* [51] proposed a coarse-grained molecular dynamics (CGMD) model for collagen molecules based on the reactive force-field simulations. Deploying this CGMD model, Depalle *et al*. [52] studied the effect of immature and mature cross-link density in a collagen fibril with a diameter of 20 nm. The simulation results highlight three different regimes of deformation during uniaxial loading of a cross-linked fibril including (i) initial uncoiling, (ii) fibril sliding, and (iii) final work hardening due to molecule stretching, prior to the final failure. A follow-up study also investigated the effect of mineral content on MCF mechanical properties [29], whereby MCFs exhibit up to five different energy dissipation mechanisms during load-deformation, including molecular uncoiling, molecular stretching, mineral/collagen sliding, molecular slippage and crystal dissociation. Even though this pioneering work facilitated novel insight into the role of minerals on the mechanical response of MCFs, it only considered a single mineralization pattern and volume. However, there is no clear consensus in the bone biomechanics community on the exact arrangement of the mineral crystals in MCFs and these features probably vary across tissues and species [12–18], which has probably resulted in the wide range of mechanical response observed experimentally in both mineralized and non-mineralized collagen fibrils [19–25]. Given the wide range of possible mineralization patterns in MCFs, there remains a limited understanding on the relative roles of mineral and collagen on both the small- and large-deformation mechanics on both mineralized and non-mineralized collagen fibrils

This study develops a CGMD framework to investigate the role of mineral arrangement on the mechanical properties of MCFs. By building on previous studies on MCFs, several case studies are considered in which the role of mineral content and mineral distribution are investigated, with particular focus on the effect of mineralization length on the load deformation response of MCFs. Through systematic investigation, a new perspective is provided on the individual role of mineral and collagen components of the MCF, which could have important implications in certain disease complications [53] where bone mineralization is altered. The information gathered in the current study is not only applicable to bone but it can be deployed in studying other soft tissues such as tendon and ligaments which are also made of collagen fibrils.

## 2. Methods

### 2.1. Coarse-grained model framework

Considering the approximately 300 nm length of a collagen molecule and low strain rates involved in quasi-static loading of collagen fibrils, the length and time scales involved in predicting the mechanical properties of fibrils are prohibitively large. Conventional molecular dynamics force-fields are unable to capture bond breaking at large deformation, while the reactive all-atom force-fields are limited in time and length scales. To reach larger simulation length and time scales, the mesoscopic model of Depalle *et al*. [52] was used, whereby collagen atoms were replaced with coarse-grained equidistant beads along the central axis of the fibril. The coarse-grained model was calibrated based on the reactive simulation of collagen molecules under several loading conditions to enable prediction of collagen behaviour under large deformation [51]. The amount of water molecules in the system affects the HAp–collagen interactions. To minimize the variation in the interaction with the amount of water molecules, the CGMD calibration was done in vacuum [29]. Since in the absence of water molecules the CGMD model was calibrated, the water molecules were not considered in the current study.

Two adjacent collagen beads belonging to the same collagen molecule interact with a bilinear bond (equation (2.1)) and a harmonic angle was considered between three consecutive beads. The bond and angle coefficients were calibrated based on a uniaxial tension simulation of single collagen molecule under a reactive all-atom force-field [51]. Based on the relevant bond style available in [52], a new bond was added to LAMMPS to model the bilinear bond interaction. The interaction between collagen beads of adjacent fibrils were modelled through non-bonded Lennard–Jones interactions (equation (2.3)), which were calibrated based on all-atom simulation of pulling one collagen fibril from two adjoining fibrils [51]. Mineral beads in the coarse-grained model used here were arranged in face-centred cubic (FCC) lattice. The interactions between the collagen and mineral beads were calculated through a Lennard–Jones potential, with coefficients calibrated with the collagen-HAp adhesion energy. A similar potential energy was used for the HAp–HAp interactions, with the HAp bulk modulus and Poisson's ratio used as parameters for the calibration [29]. Equation (2.1) describes the variables of the potential function for the bonded interaction $\left(U_{\mathrm{bond}}\right)$, small $\left(k^{\left(0\right)}\right)$ and large deformation $\left(k^{\left(1\right)}\right)$ spring constants, bead–bead distance (r), the equilibrium distances for small $\left(r_{0}\right)$ and large deformation regimes $\left({\bar{r}}_{1}\right)$, the distance at which the potential function switches $\left(r_{1}\right)$ and the cut-off distance $\left(r_{2}\right)$. The harmonic potential for angles between collagen beads is calculated with equation (2.2) in which the $U_{\theta }$, $k_{\theta }$, θ and $\theta_{0}$ represent angle potential, angle spring constant, angle value and the equilibrium angle, respectively. Equation (2.3) describes the relation between the Lennard–Jones non-bonded potential function $\left(U_{\mathrm{non}-\mathrm{bonded}}\right)$, the distance at which the potential energy is zero (σ), depth of potential well (ε), bead–bead distance (r) and the cut-off distance $\left(r_{\mathrm{cutoff}}\right)$. The parameters for equations (2.1)–(2.3) are given in table 1 and were taken from references [29,52].

2.1 $$U_{\mathrm{bond}}=\{\begin{array}{cc} \frac{1}{2}k^{\left(0\right)}{\left(r-r_{0}\right)}^{2} & \mathrm{for}\;\;\;r<\;r_{1}\\ \frac{1}{2}k^{\left(1\right)}{\left(r-{\bar{r}}_{1}\right)}^{2} & \mathrm{for}\;\;r_{1}\;\leq r<\;\;r_{2}\\ 0 & \mathrm{for}\;r>\;r_{2} \end{array},$$

2.2 $$U_{\theta }=\;\frac{1}{2}k_{\theta }(\theta -\theta_{0}{)}^{2},$$

2.3 $$\mathrm{and}\;U_{\mathrm{non}-\mathrm{bonded}}=4\epsilon \left[{\left(\frac{\sigma }{r}\right)}^{12}-\;{\left(\frac{\sigma }{r}\right)}^{6}\right]\;\;\;\;\;\;\mathrm{for}\;r<\;r_{\mathrm{cutoff}}.$$

**Table 1.** Simulation parameters in the current study taken from references [29,52].

component	parameter	value	equation
collagen	$k^{\left(0\right)}$	$17.13\;\mathrm{kcal}.{\mathrm{mol}}^{-1}.{Å}^{-2}$	(2.1)
	$k^{\left(1\right)}$	$97.66\;\mathrm{kcal}.{\mathrm{mol}}^{-1}.{Å}^{-2}$	(2.1)
	$r_{0}$	14.00 Å	(2.1)
	$r_{1}$	18.20 Å	(2.1)
	$r_{2}$	21.00 Å	(2.1)
	$\theta_{0}$	164.00–180.00°	(2.2)
	$k_{\theta }$	$14.98\;\mathrm{kcal}.{\mathrm{mol}}^{-1}.{\mathrm{rad}}^{-2}$	(2.2)
	ε	$6.87\;\mathrm{kcal}.{\mathrm{mol}}^{-1}$	(2.3)
	σ	14.72 Å	(2.3)
	$r_{\mathrm{cutoff}}$	21.00 Å	(2.3)
hydroxyapatite	ε	$193.7\;\mathrm{kcal}.{\mathrm{mol}}^{-1}$	(2.3)
	σ	10.28 Å	(2.3)
	$r_{\mathrm{cutoff}}$	13.85 Å	(2.3)
collagen-hydroxyapatite	ε	$137.1\;\mathrm{kcal}.{\mathrm{mol}}^{-1}$	(2.3)
	σ	9.88 Å	(2.3)
	$r_{\mathrm{cutoff}}$	20.00 Å	(2.3)

### 2.2. Collagen fibril structure

**Figure 1.**
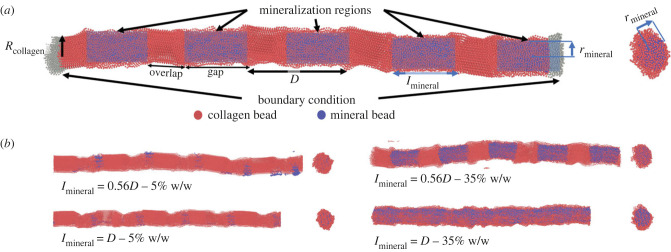
Simulation set-up. (*a*) The collagen and mineral beads are represented by dark orange and blue colours. *D*, *R*_Collagen,_ *l*_mineral_ and *r*_mineral_ are representatives of collagen periodic distance, its radius, mineralization region length and radius, respectively. (*b*) Examples of several initial systems simulated in the current study in which the degree of mineralization, and the length of the mineralization region changed.

The initial structure of the collagen molecule was adapted from the Protein Data Bank code of ‘3hr2’ [54], which is the structure experimentally resolved by Orgel *et al.* [55]. A python code was used to extrapolate the collagen central axis and obtain the coordinates of coarse-grained beads placed in 14 Å distances. Using the symmetry information provided with the structure, a collagen fibril with 20 nm diameter was built having 155 collagen molecules (e.g. protofibrils) (figure 1*a*) each with 219 beads. This size was chosen, as the simulation results of Depalle *et al*. showed that the ultimate tensile strength and tensile modulus for fibrils larger than 12 nm in diameter were independent of the diameter [52]. Also, our preliminary simulation results with diameters of 40 and 55 nm showed that the stress–strain plot is not dependent on the MCF diameter, which is in line with experimental findings of Svensson *et al*. on the diameter independency of MCF Young's modulus for diameters in 90–170 nm range [56]. Accordingly, the 20 nm diameter MCF simulated in the current study provides a basis to study mechanical properties of biological MCFs with diameters in 20–500 nm range [57]. Our preliminary simulation results showed that, increasing the MCF length, the effect of mineral amount on the MCF mechanical properties changes until the length of 30*D* (2.01 µm) after which the variation in MCF mechanical properties with mineral contents remains the same. Accordingly, for the simulations on the effect of mineralization amount, the MCF length was chosen as 50*D* (3.35 µm) (§3.1). However, the simulation results showed that after length of 5*D* (0.334 µm), the influence of the mineral distribution on the MCF mechanical properties remains qualitatively the same with larger increase in the mechanical properties. To make simulations on the mineralization region length computationally feasible (§3.2) length of 5*D* (0.334 µm) was chosen. Immature collagen fibrils without cross-links were considered in the current study to separate the effect of mineralization on the MCF mechanical properties from the cross-linking effect.

### 2.3. Mineralization patterns

To build the MCFs, mineral beads were introduced to the fibril into a mineralization region, whose length was varied to obtain various mineralization patterns (figure 1*a*). Initially, the whole mineralization region was filled with HAp beads. Then, any HAp beads that were closer than a cut-off value to collagen beads were removed using a script in the VMD software package [58]. The cut-off value was chosen through a trial-and-error process to provide the desired mineral amount (electronic supplementary material, table S1). Then, the coordinates of collagen and mineral beads were fed into a C++ code to write the LAMMPS input file. In the current study, different degrees of mineralization of 5% and 35% w/w were considered having approximately 36 000 and approximately 51 000 beads, respectively. A non-mineralized fibril was also considered (0% w/w).

In the simulation sets considered here, the effect of mineralization region length on the mechanical properties of MCFs was examined. Here, the mineral radius was equal to the initial MCF radius (10 nm), while four different mineralization region lengths of $l_{\mathrm{Mineral}}=(0.44,\;\;0.56,\;\;0.77,\;\;1)D$ were considered, where *D* is the collagen fibril periodic length. For different mineral lengths considered, the shortest mineral $l_{\mathrm{Mineral}}=0.44D$ had a mineralization region that minerals only resided in the gap region, while the longest mineral $l_{\mathrm{Mineral}}=D$ had a mineralization region that extended from the gap region fully into the overlap region. The other cases of $l_{\mathrm{Mineral}}=0.56D$ and $l_{\mathrm{Mineral}}=0.77D$ had mineralization regions that extended into 0.2 of the overlap length and 0.6 of the overlap length, respectively (figure 1*b*). For the degree of mineralization of 5% w/w, the mineralization lengths of $l_{\mathrm{Mineral}}=0.44D,0.56D,0.77D\;\mathrm{and}\;D$ lead to the overlap mineral concentration of 0%, 0.58 ± 0.07%, 1.17 ± 0.04% and 1.61 ± 0.03% w/w, respectively, while the corresponding values for the 35% w/w degree of mineralization were 0%, 4.40 ± 0.38%, 10.14 ± 0.33% and 13.73 ± 0.33% w/w. Each simulation was repeated at least five times giving rise to the total number of 192 simulations (electronic supplementary material, table S1) with the cumulative simulation time of 40 µs.

### 2.4. Uniaxial tension simulations

A uniaxial tension test was simulated to study the load-deformation response of the MCFs with different degrees of mineralization and patterns. Before the main simulation, an equilibrium simulation in constant number of particles, pressure and temperature (NPT) ensemble of 1 bar and 300 K was carried out for a duration of 20 ns to relax the MCFs, which has been found to be sufficiently long to reach the equilibrated state [29]. The Nosé–Hoover thermostat with equations of motion as obtained by Shinoda *et al*. [59] was used in the equilibration step and the pressure was kept constant only in the z-direction while during uniaxial loading, done with the Nosé–Hoover thermostat in constant number of particles, volume and temperature (NVT) ensemble, one end-region of the MCF corresponding to 2.5% of its total length was pulled with a fixed velocity to induce a constant strain rate and the other end-region having the same length was kept fixed. The secant modulus was calculated as the stress–strain ratio at strain of 0.04. The toughness was defined as the area under the stress–strain curve until the final fracture event. A parametric study showed that the strain rate of $2.5*10^{6}\;s^{-1}$, used in the current study, led to fracture in the central region of the MCFs. This value is four times lower than the strain rate of $10^{7}\;s^{-1}$ adopted by Depalle *et al*. [29], which is possibly due to different mineral distribution pattern. Considering the initial length of 300 nm for the collagen molecule, the chosen strain rate meant a pulling rate of 0.75 m s^−1^ was applied. This pulling rate was lower than the 1 m s^−1^ pulling speed threshold below which the Young's modulus of collagen molecule is rate-independent according to Gautieri *et al*. [60]. The stress–strain curves for 0%, 5% and 35% w/w mineralized MCFs with minerals only in the gap region were in good agreement with the results obtained in [29]. The virial stress was calculated according to equation (2.4), in which the integration is carried out over $N_{p}$, $N_{b}$ or $N_{a}$ neighbours of atoms i, its bonded neighbours or the atoms with which it has angle interactions. The $S_{\mathrm{ab}}$, $r_{1a}$, $r_{2a}$, $\left(r_{3a}\right)$ represent the ab component of the virial stress tensor and the positions of two atoms (three atoms) in pairwise (angle) interactions. Summing the virial stress values [61] over all the beads and dividing them by the current MCF volume, the effective stress was obtained. In calculating the stress values of each individual MCF component, its volume was considered proportional to its fraction of total beads assuming an equal volume for different bead types. To study the protofibril stress distribution, sliding and scaled centre of mass values, several python codes were developed which are included in the electronic supplementary material. LAMMPS software was used for the molecular simulations [62] and the visualizations were done with the Matplotlib package of python [63] and OVITO software [64].

2.4 $$S_{\mathrm{ab}}=\;\frac{1}{2}\overset{N_{p}}{\underset{n=1}{\sum }}(r_{1a}F_{1b}+r_{2a}F_{2b})+\;\frac{1}{2}\overset{N_{b}}{\underset{n=1}{\sum }}(r_{1a}F_{1b}+r_{2a}F_{2b})+\;\frac{1}{3}\overset{N_{a}}{\underset{n=1}{\sum }}(r_{1a}F_{1b}+r_{2a}F_{2b}+r_{3a}F_{3b}).$$

## 3. Results

### 3.1. Effect of mineralization on MCF mechanical properties

**Figure 2.**
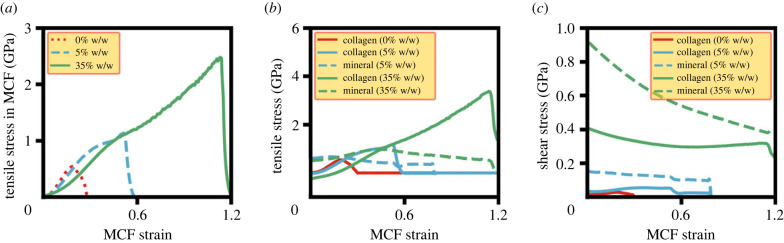
Mechanical properties of MCF with various degrees of mineralization. (*a*) Stress–strain curves for MCFs. (*b*) Tensile and (*c*) shear stress for collagen and mineral phases of MCF plotted against MCF strain.

Figure 2*a* shows the stress–strain response for MCFs with three different degrees of mineralization of 0%, 5% and 35% w/w, in which only the gap region is mineralized with the radius of the mineralization regions (*r*_Mineral_) equal to the collagen fibril radius (*R*_Collagen_) (figure 1). Two different mineralization regimes of slightly (5% w/w) and highly (35% w/w) mineralized MCFs show distinct differences in mechanical behaviour compared with the non-mineralized fibril (figure 2*a*). The mineralized MCFs show more work-hardening taking place and higher values of UTS, fracture strain and toughness for increasing levels of mineralization. The post-yield behaviour of the non-mineralized fibril shows a gradual decrease in stress, suggesting ductile fracture behaviour, while for the highest level of mineralization (35% w/w), there is a sudden drop in tensile stress once the UTS is reached.

**Figure 3.**
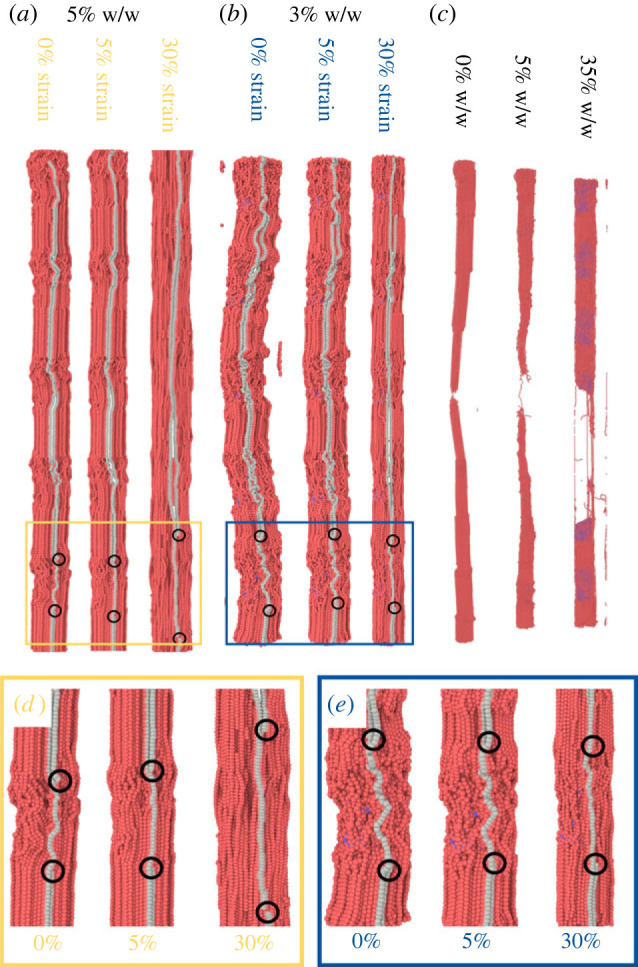
(*a*) The simulation snapshots for MCF with degrees of mineralization of 5% w/w in different MCF strains shows the sliding for two protofibrils coloured differently to the rest of protofibrils. (*b*) The simulation snapshots for MCF with mineralization degree of 35% w/w in different MCF strains does not show the protofibril sliding. (*c*) The final fracture snapshots for MCFs with various degrees of mineralization. (*d*) Zoomed view of panel (*a*) related to the 5% w/w case under various strains shows the protofibril sliding. (*e*) Zoomed view of panel (*b*) does not show protofibril sliding. In these snapshots the first and last beads of one of the protofibrils are shown with circles to clarify the sliding of this protofibril with respect to the other protofibril.

To better understand the individual roles of the MCF constituents, the tensile stresses of collagen and mineral beads of MCFs were calculated and plotted against the overall MCF strain in figure 2*b*,*c*. For both the degrees of mineralization of 5% and 35% w/w, the mineral tensile stress is larger than the corresponding value of the collagen when the overall strain regime is low (less than 15%), as shown in figure 2*b*. Thus, the largest portion of the external loading is carried by the mineral phase for low strains. However, for larger strains, the collagen protofibrils accommodate most of the tensile stress in the MCF (figure 2*b*). Here, the shear stress provided by the mineral beads (figure 2*c*) reduces the amount of collagen sliding (figure 3*a,b*), which readily takes place at large strain (greater than 15%) in the non-mineralized collagen (0% w/w). Lower levels of collagen sliding in the large strain regime in MCFs increases the tensile stress on the collagen protofibrils,^1^ and this ultimately leads to unwinding and stretching of unwound collagen fibrils [47], which means that the collagen stress exceeds that of the mineral beads.

Increasing the degree of mineralization, the tensile stress carried by the collagen phase increases (figure 2*b*) as a consequence of higher mineral shear stress (figure 2*c*). Due to lower mineralization in the 5% w/w case, the mineral shear stress is lower (figure 2*c*), and there is protofibril sliding near the UTS strain (figure 3*a*,*d*) that reduces the collagen stress value at UTS compared with the corresponding value for 35% w/w (figure 2*b*). For the highest mineralization (35% w/w), there is a higher shear stress (figure 2*c*) because of larger number of minerals that eliminate protofibril sliding (figure 3*b*,*e*), ultimately leading to a larger external stress on MCF (figure 2*a*) and a different fracture behaviour (figure 3*c*).

Thus, in small deformation, external loading is primarily carried by the mineral, while deformation is mainly a result of collagen straightening. At large deformation, the collagen protofibrils which are straightened have a reduced or eliminated sliding due to the presence of minerals, and they are primarily responsible for bearing the external load. These features are clearly evident in figure 3*a*,*b* where simulation snapshots of individual protofibrils show straightening at different strain levels, lower protofibril sliding for higher degrees of mineralization (figure 3*b*) and a different fracture behaviour (figure 3*c*).

**Figure 4.**
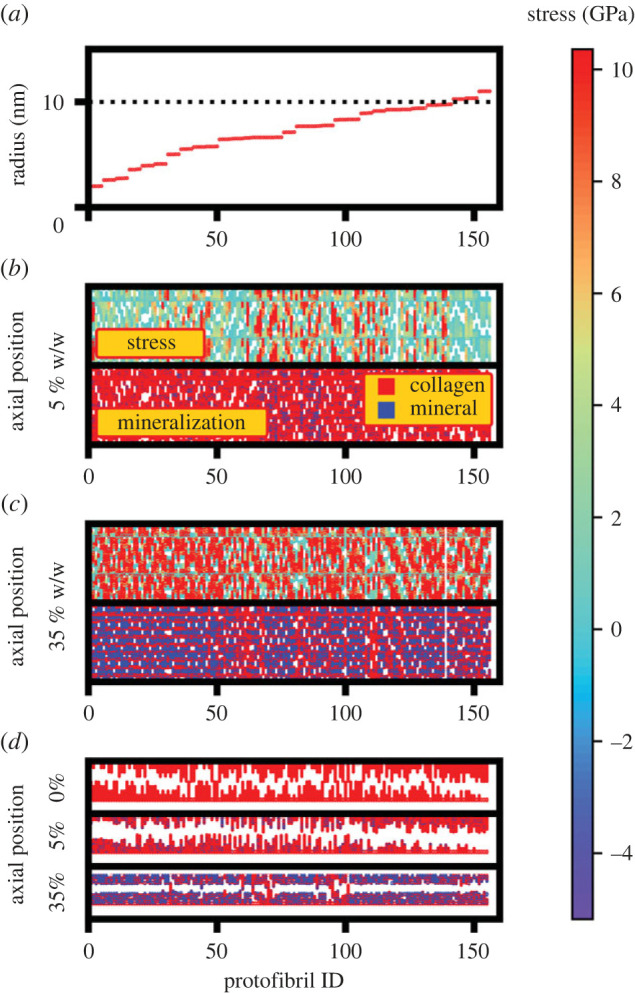
The role of different collagen protofibrils in uniaxial loading of MCFs with various degrees of mineralization. (*a*) The average distance from the MCF central axis (i.e. protofibril radius) for different protofibril IDs with higher protofibril IDs assigned to collagen molecules with larger radius values. The MCF radius is shown with dotted line. The stress distribution (upper panel) and mineralization distribution pattern (lower panel) for various collagen protofibrils with (*b*) degrees of mineralization of 5% w/w and (*c*) 35% w/w showing higher stress values in mineralized protofibrils. In the lower panel mineralized and non-mineralized protofibrils are coloured blue and red. (*d*) The protofibril axial position for MCFs with various degrees of mineralization at the fracture point illustrating the sliding of non-mineralized protofibrils impeding with the cracks initiated from their mineralized counterparts.

To reveal the MCF stress distribution, protofibril IDs were assigned to the collagen beads, with higher values allocated for those belonging to protofibrils with larger radial location in the fibril^2^ (figure 4*a*). The protofibril stress distribution at UTS represents a non-uniform distribution for 5% w/w (figure 4*b* upper panel) with the external loading mainly exerted to the mineralized protofibrils (figure 4*b* lower panel). This is similar for the 35% w/w case (figure 4*c*) at UTS. Comparing the protofibril stress distribution for these two cases (figure 4*b*,*c*), an increase in the mineralization results in a greater number of protofibrils under loading, and this ultimately contributes to its higher UTS value (figure 2*a*). The axial position for different protofibril IDs at the fracture point for degrees of mineralization of 5% and 35% w/w (figure 4*d*) show that fracture behaviour does depend on the mineral content. For the non-mineralized fibrils, the variation in the collagen beads' z-coordinate, with their protofibril ID demonstrates that protofibril sliding is the underlying reason for fracture events, predicting purely ductile fracture behaviour. Increasing the mineral percentage to 5%, the axial position plot shows crack initiation from the mineralized protofibrils, with sliding of non-mineralized protofibrils acting as a barrier to the crack propagation. Increasing the mineralization degree to 35% w/w, the final crack size is larger for higher degrees of mineralization, as there are more mineralized protofibrils and the number of non-mineralized protofibrils responsible for crack deflection reduces. Thus, non-mineralized protofibrils in the MCF are responsible for arresting crack propagation through sliding, while their mineralized counterparts carry the external loading (further explanation of this observation is available in the electronic supplementary material, text).

### 3.2. Effect of mineralization length on MCF response

**Figure 5.**
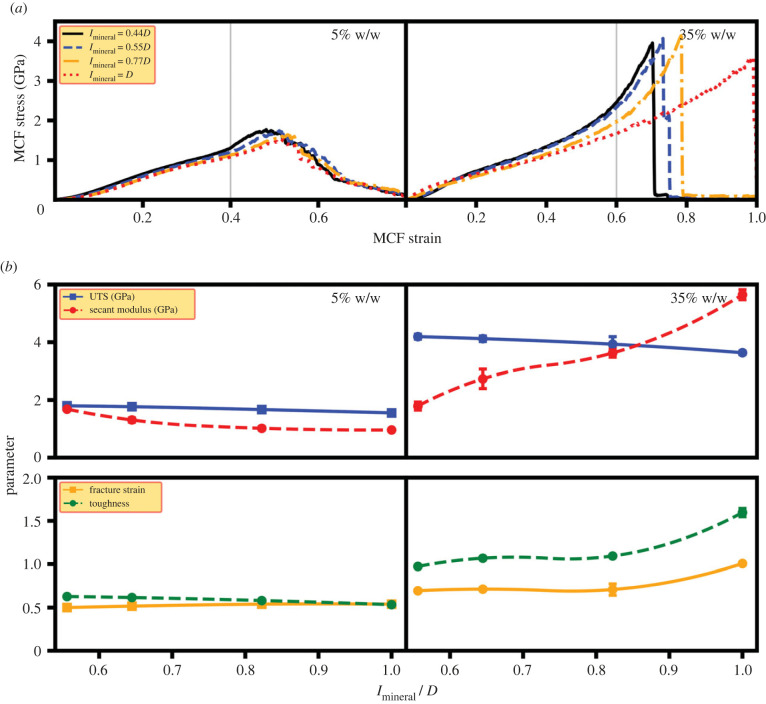
Mechanical properties of MCFs with different mineralization lengths. (*a*) The stress–strain plots and (*b*) The mechanical properties of different mineralization lengths. ‘*D*’ illustrates the periodic length of collagen fibril with *l*_mineral_/*D* = 0.56, *l*_mineral_/*D* = 1 representing an MCF with minerals in the gap region and MCF mineralized in its whole length, respectively. The lines in the panel (*b*) just serve as a guide to the eye.

Figure 5 shows the predicted mechanical properties of MCFs for several mineralization lengths, simulated for both the degrees of mineralization of 5% and 35% w/w. The predicted stress–strain responses are shown in figure 5*a*, where the mineralization region length has little impact on the mechanical properties for the 5% w/w case. On the other hand, for its highly mineralized counterpart, there is a decrease in UTS while the secant modulus shows a substantial increase for higher mineral lengths, with higher toughness and fracture strain for longer mineralization region.

**Figure 6.**
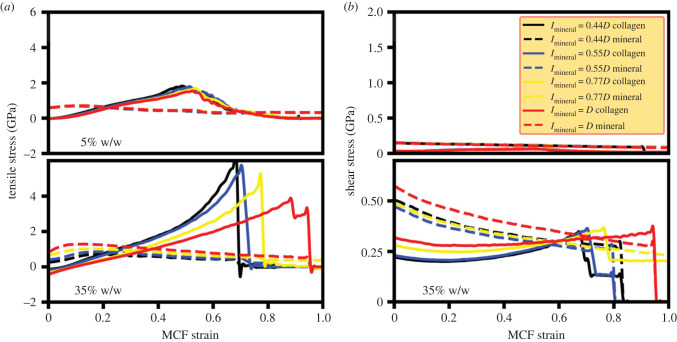
The role of the collagen and mineral phase of the MCF in the uniaxial loading of the MCF. (*a*) Tensile and (*b*) shear stress of collagen and mineral phase for different mineralization lengths.

The stress–strain plots for the collagen and mineral components of MCFs with different mineralization lengths are shown in figure 6 and are the average values obtained from at least three simulations (see electronic supplementary material, table S1). These plots show similar behaviour to those described in §3.1 but provide additional insight into the role of constituents in dictating the load-deformation response. Here, non-zero values of tensile stress in the absence of applied strain demonstrate the residual stress state observed in the collagen and mineral stress–strain curves for various mineralization regimes. The residual stress remains the same for the mineralization degree of 5% w/w across various mineralization lengths, while it increases with the mineralization region length for the mineralization degree of 35% w/w. The increase in the residual stress with increasing the mineralization length for the 35% w/w case leads to an increase in the stress carried by the mineral phase in the small deformation regime. Thus, at small deformation regimes, the mineral phase carries higher stress for larger mineralization lengths. Considering the larger modulus of HAp, there is an increase in the secant modulus with increasing the mineralization length of 35% w/w (figure 5).

Also, a toe-region in the stress–strain plots is observed (figure 5) whose size remains the same for 5% w/w across mineralization regimes, while it decreases in size with increasing the mineralization length for 35% w/w. The size of the toe-region for 5% w/w is almost independent of the mineralization region length owing to the very low mineral percentage of this case (figure 6*a*). However, for 35% w/w, the toe-region starts at the MCF strain of zero and its endpoint coincides with where the load carried by the mineral increases (figure 6*a*).

The stress–strain plots for the shear stress of individual MCF components shows lower levels of shear stress for 5% w/w (figure 6*b*), which results in its larger protofibril sliding. For the 35% w/w case, the large shear stress on the collagen and mineral phases impeded protofibril sliding and facilitated load-transfer between these constituents. Also, the collagen and mineral shear stress at the fracture point is the same for different mineralization lengths (figure 6*b*) despite their different tensile stress (figure 6*a*), which suggests a shear fracture mode.

**Figure 7.**
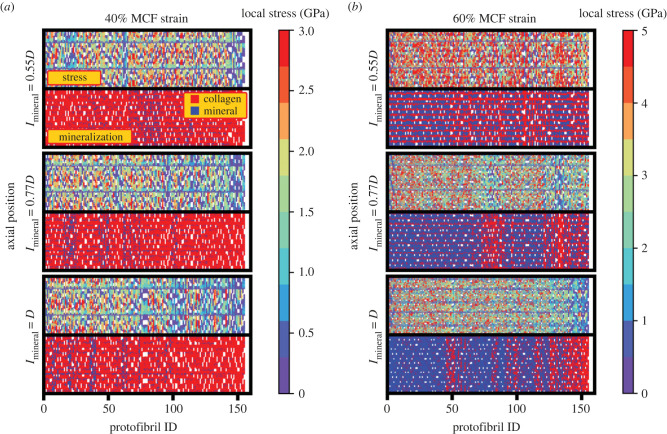
The scaled protofibril axial position–ID plots for different protofibrils across various mineralization lengths. The plots are coloured based on the stress values and mineralization status in the upper and lower panel, respectively. Degrees of mineralization of (*a*) 5% w/w and (*b*) 35% w/w. The graphs not only confirm the role of mineralized protofibrils in carrying the external loading, but they also provide an explanation on the decrease in the MCF stress with increasing the mineralization length in a fixed strain value.

Figure 7 presents the stress and mineralization distribution profiles for MCFs with both the 5% and 35% w/w cases, plotted at a total MCF strain of 40% and 60%, respectively (corresponding to the strain of the grey lines in figure 4). The upper subpanel is coloured based on the local stress values with the colour bar showing the magnitude of tensile stress. In the lower subpanel, each protofibril bead is coloured based on its mineralization status with the mineralized protofibrils coloured blue and non-mineralized beads coloured red. Comparing the upper with lower subpanel for each case, it is evident in the 35% w/w case that beads with larger stress values (red coloured in the upper subpanel) are the same as the mineralized beads (blue coloured beads of the lower subpanel). Thus, the mineralized collagen protofibrils carry the external loading in the highly mineralized MCF.

Figure 7 also explains why MCF stress values decrease with increasing the mineral length, which was seen by the stress values along the grey line in figure 5*a*. For the lower mineralization case of 5% w/w, there is a minor effect of mineralization length on the stress distribution (figure 7*a*). However, for the highly mineralized case of 35% w/w, both the decrease in the number of mineralized protofibrils and the reduction in the stress carried by each protofibril (figure 7*b*) resulted in a reduction in the total stress, with increasing the mineralization length. The reduction in the number of mineralized protofibrils is due to the arrangement of minerals in smaller radius for larger mineralization lengths. Increasing the mineralization length, the residual strain increases, which in turn leads to a lower effective strain on each protofibril at a specific external MCF strain. Thus, the stress on each protofibril is lower causing a decrease in the MCF stress at a specific strain with increasing the mineralization length.

**Figure 8.**
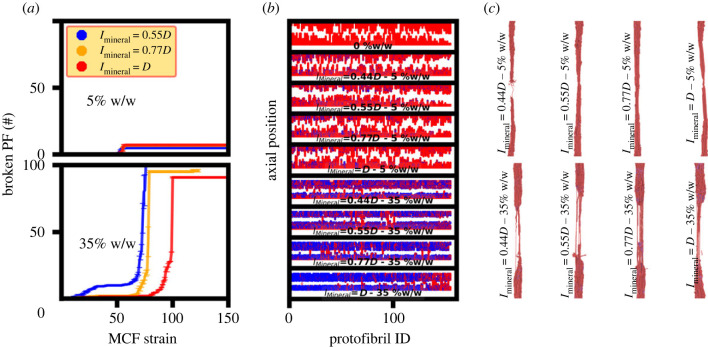
Fracture behaviour of MCFs across various mineralization lengths explaining the trend observed for the UTS values and also the role of non-mineralized protofibrils in stopping the cracks initiated from their mineralized counterpart. (*a*) The number of broken protofibril – MCF strain for various mineralization lengths showing more gradual breaking for larger mineralization lengths of 35% w/w. (*b*) The axial position – protofibril ID values for various mineralization lengths and several degrees of mineralization showing the sliding of non-mineralized protofibrils as the reason for stopping the cracks initiated from their mineralized counterpart. The non-mineralized and mineralized protofibrils are colored red and blue, respectively. (*c*) The fracture snapshots for MCFs with different degrees of mineralization and mineralization patterns.

The mineral distribution affects the UTS value. The protofibril stress distribution for both the degrees of mineralization of 5% and 35% w/w at the UTS strain illustrates higher stress values for the mineralized protofibrils and also a decrease in the stress carried by mineralized protofibrils of 35% w/w with increasing the mineralization length (electronic supplementary material, figure S6). Figure 8*a* shows that there is a lower number of broken protofibrils in the 5% w/w compared with the 35% w/w. Also, more gradual protofibril breaking for larger mineralization lengths of 35% w/w results in lower work hardening and a decrease in the UTS with increasing mineralization length for this case. Gradual protofibril breaking is caused by a more non-uniform protofibril stress distribution at UTS for larger mineralization lengths of 35% w/w (electronic supplementary material, figure S6).

The axial position–protofibril ID graphs for several MCFs across a range of degree of mineralization and mineralization region lengths all show MCF fracture in the mineralized protofibrils (blue beads in figure 8*b*), with their non-mineralized counterparts stopping crack propagation (red beads in figure 8*b*), confirming the previous observation on fracture behaviour. The fracture snapshots also confirm this observation (figure 8*c*). An increase in the mineralization length for a fixed degree of mineralization (figure 8*b*) leads to an increase in the number of crack deflecting protofibrils. Comparing the fracture snapshots of 35% w/w (figure 8*c*) for *l*_Mineral_ = 0.44*D* (in which the minerals are only in the gap region) with its counterpart of *l*_Mineral_ = *D* (minerals in the whole protofibril length), it is observed that the increase in the mineralization length causes an increase in the number of crack deflecting non-mineralized protofibrils. The mineralization length has less influence on the fracture mode of 5% w/w probably owing to its lower mineralization percentage and higher protofibril sliding.

**Figure 9.**
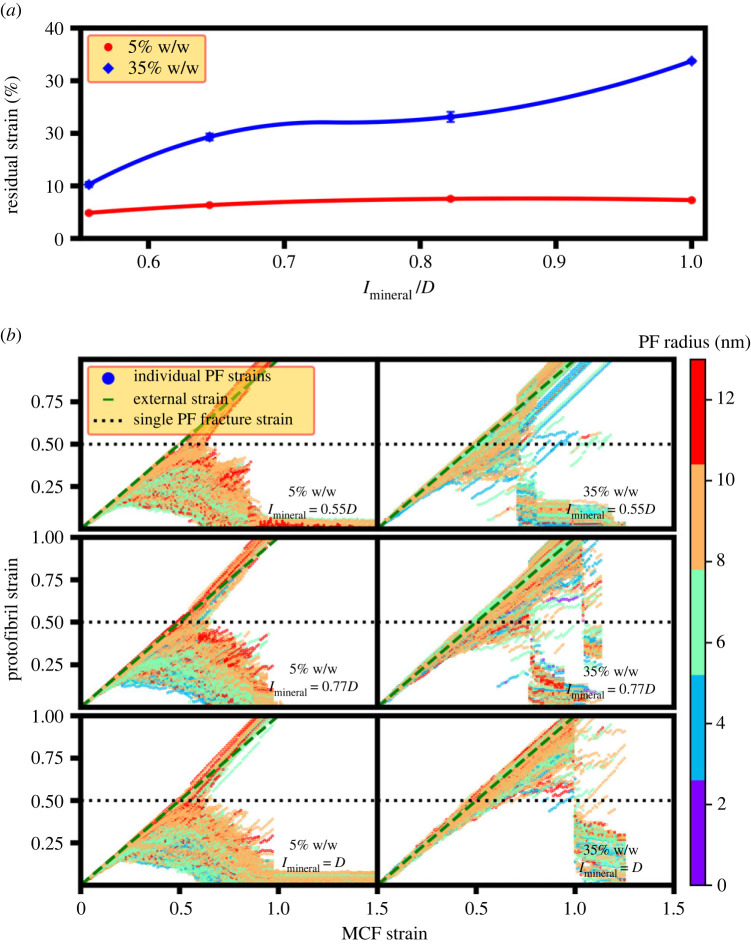
Strains in MCFs with different degrees of mineralization and mineralization lengths. (*a*) MCF residual strains for degrees of mineralization of 5% and 35% w/w with different mineralization mineralization lengths. (*b*) Tensile strain for individual protofibrils of MCF with different degrees of mineralization and various mineralization length values.

For both the degrees of mineralization of 5% and 35% w/w, there is an increase in the residual strain with increasing the mineralization length (figure 9*a*), which is the reason why the fracture strain increases (figure 5*b*). Also, the higher fracture strain of 35% w/w than 5% w/w is a consequence of its larger residual strain. The protofibril strain distribution for 5% w/w (figure 9*b*) shows that the broken protofibrils all break at the fracture strain of a single collagen molecule [65] implying that the increase in the residual strain is the primary reason for its fracture strain trend.^3^ For 35% w/w, however, the protofibril fracture strain is higher for the larger mineralization length (figure 9*b*) which provides another explanation for its increased fracture strain.

Quantitative analysis of the protofibril movement shows that the drop in the tangent modulus in the stress–strain plot (figure 5*a*) at the end of the toe-region corresponds to the start of protofibril sliding (electronic supplementary material, figure S2). From changes observed in the protofibril centre of mass distribution (see electronic supplementary material, figure S2) with mineralization length, it is implied that the sliding occurs in the non-mineralized protofibril length in agreement with previous studies [39]. It is also observed that higher protofibril straightening in the toe-region leads to higher subsequent sliding and more uniform protofibril stress distribution afterwards. The occurrence of protofibril sliding after the straightening is in agreement with previous experimental findings on tendon mechanics [66]. More information on the protofibril straightening and sliding are presented in the electronic supplementary material, information.

## 4. Discussion

This study implemented a coarse-grained molecular dynamics (CGMD) framework to investigate the role of mineralization on the mechanical properties of MCFs. Interestingly, our results demonstrated that the mineral phase played an important role at large deformation by facilitating shear stress transfer between collagen molecules, which inhibited collagen sliding and enabled greater work-hardening to take place. The effect was enhanced with increasing mineralization. However, while reduced collagen sliding enhanced MCF properties, our simulations demonstrated that complete elimination of protofibril sliding was not desirable as it led to reduced ultimate tensile strength (UTS) and work hardening. It was found that the mineralization patterns also affected mechanical properties, although to a lower extent than overall mineral content. Depending on the mineralization pattern, we observed that there were distinct roles for protofibrils within MCFs, with mineralized protofibrils carrying the external loading, while non-mineralized protofibrils playing an important role in deflecting local cracks within the fibrils. These results provide novel insight into the load-deformation behaviour of MCFs and highlight the intricate role that both collagen and mineral components have in dictating higher scale bone biomechanics.

MCFs can be considered as one of the universal building blocks of various mineralized tissues such as bone and tendon [3,4]. Characterizing their mechanical properties can help understand the structural role of such tissues through multi-scale or homogenization approaches [26–28]. In experimental studies on mineralized and non-mineralized collagen fibrils, a wide range of mechanical properties have been reported, with fracture strains ranging between 5.96% and 106.55% and tensile strengths between 39 and 1000 MPa, respectively [19–25]. The wide variation in properties is possibly due to variation in the mineralization amount and pattern between collagen fibrils isolated from various sources. For non-mineralized collagen fibrils, our model predicted a fracture strain of 24.6%, which showed good agreement with the value of 23% experimentally measured by Gentleman *et al*. for rat tail tendon [20]. Our predicted UTS value of 543 ± 1 MPa was several times larger than that found by Gentleman *et al.* [20], which is possibly due to the lack of solvation in the current study. However, our measured UTS showed better agreement with Liu *et al*. who determined a UTS of 638 ± 98 MPa for dry collagen fibrils from calf skin [19]. Our results showed that a small increase in mineralization to 5% w/w almost doubled the fracture strain and UTS values, with the increase in these parameters continuing for higher amounts of mineral (35% w/w). These findings appear to contradict the experimental results of Hang & Barber [21] in which the fracture strain from antler bone was only in the range of 5.14–6.72%. However, it should be noted that the single MCF being simulated in our study represents a highly idealized system, which is difficult to physically isolate and test in the absence of any extrafibrillar mineral, and therefore experimental comparisons might not be directly applicable. It is worth noting that the predicted results from our baseline MCFs showed agreement with computational predictions by Depalle *et al*. [52], who considered a similar isolated MCF representation. With this in mind, our results provide novel insight into the mechanical response of this fundamental tissue unit, considering it as an isolated system. One of the primary reasons for higher fracture strains of the MCFs with increasing mineral content was the higher residual strains in the system caused by presence of these minerals, which is a feature that has also been observed experimentally [67]. Eliminating the residual strain, our simulations showed that the fracture strain actually remained unchanged for different mineral contents. For MCFs that included residual strains, it was found that higher levels of mineralization eliminated the sliding of collagen molecules, which enabled them to undergo tensile loading, thereby providing a substantial contribution to the UTS of MCFs, while facilitating higher fracture strains. These predicted properties demonstrate that MCFs could potentially dissipate large amounts of energy in the tissue microstructure, although this behaviour was observed in a deformation regime that was well beyond typical tissue fracture strains. With the surrounding extrafibrillar matrix in bone, this perhaps raises the question as to when this large deformation behaviour of MCFs would be observed, or even required. However, there may be certain scenarios during fracture events where MCFs could operate as isolated systems. In particular, several experimental studies on bone tissue have observed that fibrillar/fibre structures provide an extrinsic toughening mechanism to cracks that are propagating. Therefore, it could be the case that the excellent capacity for energy dissipation we have observed here for MCFs may become activated once fracture of the extrafibrillar matrix has taken place, with these structures undergoing large-deformation and providing additional toughening through this bridging mechanism. Future computational studies should consider the presence of this extrafibrillar matrix to better understand the typical tissue-level performance of the lamellar bone system.

Previous studies have suggested that the role of the mineral phase in MCFs was to provide stiffness, while collagen molecules were responsible for increasing the structure's flexibility [3,29,32]. Our results identified these same roles for the constituent phases of MCFs (figure 2), however, only in the cases of small deformation regimes (MCF strain less than 15%). For the large deformation regime, our results show a distinct role for the collagen phases in load carrying, with tensile stresses in collagen much higher than the mineral phase (figure 2*b*). This was observed across all levels of mineralization and each of the 30 different mineralization patterns that were investigated. Here, we identified that the mineral phase was primarily responsible for facilitating load take-up by collagen molecules, where its role was to transfer the external loading between adjacent collagen protofibrils at large deformation. For higher levels of mineralization, the mineral shear stress increased, as did the capacity of the collagen phase to carry larger tensile stress, explaining the enhancement in the MCF mechanical properties observed here for larger degrees of mineralization (figure 2*a*).

While experimental and theoretical studies have widely acknowledged that protofibril sliding takes place in the collagen molecule [29,52,68–73], there has been limited quantitative investigation of this phenomenon to date due to the experimental resolution required to capture this mechanism and prohibitive computational cost. Through development of new computational tools, this study provides detailed insight into the mechanisms responsible for collagen protofibril sliding and its effect on MCF properties. Here, we show that fibril sliding occurs after the initial fibril straightening, which is in agreement with the experimental observations by Folkhard *et al.* [73]. The mineral phase is responsible for restricting the amount of collagen protofibril sliding, through transfer of shear stress between adjacent protofibrils. While lower protofibril sliding enhanced the mechanical properties of MCFs, the simulation results across various mineralization patterns showed that a certain amount of sliding was still necessary to ensure a uniform protofibril stress distribution and enhanced mechanical properties. To be more specific, the residual strains are non-uniformly distributed among the protofibrils, which leads to a non-uniform protofibril stress distribution when there is a lack of protofibril sliding. If the mineral eliminates protofibril sliding completely, gradual protofibril breaking occurs that ultimately results in a lower amount of work hardening. Even though reduced protofibril sliding is the mechanism through which the mineral enhances the mechanical properties of MCFs, a certain amount of sliding is required for the partial release of the non-uniform protofibril residual strain to ensure work hardening can take place. This study is the first to establish these mechanisms by which protofibril sliding is responsible for MCF mechanical properties, building on the work of Depalle *et al*. [29], and identifying for the first time how shear transfer between adjacent protofibrils results in substantial work hardening in MCFs.

Currently, there is no clear consensus in the bone community on the precise distribution of mineral in MCFs [8–18]. In early studies, it was suggested the mineral resided in the gap region due to a lower collagen density [8–10]. However, further assessment of bone mineral density [11] and more detailed discovery of the MCF structure [48] identified that there was insufficient space in the region to accommodate overall mineral content present in the tissue. As a consequence, it was suggested that the mineral must reside outside the gap region or even in the extrafibrillar space [12–18]; however, there have been few investigations on the role of mineralization pattern on the mechanical properties of MCFs. Our study explored 30 different mineralization patterns and showed that mineralization beyond the gap region resulted in higher secant modulus, residual strain and toughness, while it slightly decreases the UTS. It was found that the mineralization pattern with the highest UTS had greatest proportion of mineralized protofibrils, which highlights the importance of distinguishing between mineralized and non-mineralized protofibrils within the MCF. The effects of these mineralized and non-mineralized regions are seen almost immediately in the stress–strain plots, whereby protofibril straightening was observed in the non-mineralized protofibril lengths in the toe region in agreement with previous studies [74,75] which facilitated subsequent protofibril sliding. A longer mineralized length led to a smaller non-mineralized length, which limited the amount of straightening that could occur. Thus, a longer mineralization length underlies the non-uniform protofibril stress distribution by preventing protofibril straightening, which leads to more gradual protofibril breaking, a decrease in the UTS and lower protofibril sliding. Upon crack propagation, it was generally observed that mineralized protofibrils within MCFs carried the external loading, while their non-mineralized counterparts were responsible for arresting crack propagation through sliding. Due to distinct roles for mineralized and non-mineralized protofibrils in MCF, the simulation results showed that their specific arrangement across different mineralization patterns lead to different mechanical properties, with mineral distribution in a wider spatial arrangement causing lower UTS and higher ductility.

Thus, the current study has provided novel insight into the role of mineralization on the load-deformation behaviour of MCFs. However, there are some limitations in the current study. Firstly, Lennard–Jones potential functions were used for both the collagen–mineral interface and mineral–mineral interactions. As the simulations showed, the interface has a significant influence on the load transfer between the MCF components, and further molecular simulation studies with all-atom resolution would be required to obtain better interface parameters for this force-field. Furthermore, in this study, we only consider a single MCF and do not consider other components of the ultrastructural arrangement. In particular, the extrafibrillar matrix and non-collagenous proteins, which are shown to significantly affect the bone mechanical properties [40–43,76,77], have not been considered. Several other studies have explicitly considered the important role of extrafibrillar mineralization on the effective properties of the tissue [40–44], and these have shown excellent agreement with experimental observations in the elastic regime. However, the model presented here is an idealized MCF model, which is more difficult to validate as it cannot be physically isolated from the tissue in the absence of an extrafibrillar mineral. Still, the findings presented have provided novel insight into the mechanical performance of these important building blocks of mineralized and non-mineralized tissues. Future studies will consider a more complex extrafibrillar matrix to fully understand bone biomechanics at this scale.

## 5. Conclusion

A coarse-grained molecular dynamics (CGMD) framework was developed to investigate the mechanical properties of mineralized collagen fibrils (MCFs). The simulation results for different degrees of mineralization showed a role for minerals in enhancing the MCF mechanical properties. Specifically, in the large deformation regime, it was found that the mineral phase was responsible for limiting collagen sliding through high shear stress, which helped the collagen molecules themselves undergo high tensile loading, providing a substantial contribution to the UTS of MCFs. However, the simulation results showed that a certain amount of collagen sliding was still necessary to ensure a uniform protofibril stress distribution and prevent early protofibril fracture. It was found that the mineral distribution affected the MCF mechanical properties to a lower extent than the overall mineral content. This study also highlights different roles for the mineralized and non-mineralized protofibrils within the MCF, with the mineralized groups responsible for loading carrying due to the presence of the mineral phase, while the non-mineralized counterparts play an important role in crack deflection. Together, the results from this study provide novel insight into the load-deformation response of MCFs, which form a universal building block of mineralized tissues.

## Data Availability

All the files necessary to reproduce the results reported here alongside with the processed simulation data are openly available in Figshare at https://doi.org/10.6084/m9.figshare.20109110.v1. The data are provided in electronic supplementary material [78].

## Endnotes

1 In highly mineralized MCFs, debonding probably happens at the interface of mineralised and non-mineralized collagen protofibrils which is outside the scope of the current study.

2 Here, a bead radius is defined as its distance from the MCF central axis.

3 The results for this case also show that the protofibrils having maximum strain switch from higher radius value protofibrils to those with lower radius with increasing the mineralization length, which is in line with (i) the findings of the previous sections in the role of the mineralized protofibrils on carrying the external loading and (ii) the distribution of minerals in smaller radius values for longer mineralization lengths.
